# Molecular mechanisms from reaction coordinate graph enabled multidimensional free energies illustrated on water dimer hydrogen bonding

**DOI:** 10.1002/jcc.26982

**Published:** 2022-08-22

**Authors:** Tomás Bruce‐Chwatt, Kevin J. Naidoo

**Affiliations:** ^1^ Scientific Computing Research Unit, Department of Chemistry University of Cape Town Cape Town South Africa

**Keywords:** AIMD, density functional theory, free energy, graph theory, hydrogen bonding, water dimer

## Abstract

Computing the free energies of molecular mechanisms in multidimensional space relies on combinations of geometrically complex reaction coordinates. We show how a graph theory implementation reduces complexity, and illustrate this on the arrangements of hydrogen bonding of a water dimer. The reaction coordinates and forces are computed using graphs that define the dependencies on the atoms in the Free Energy from Adaptive Reaction Coordinate Forces (FEARCF) library. The library can be interfaced with classical molecular dynamics as well as quantum molecular dynamics packages. Multidimensional interdependent reaction coordinates are constructed to produce complex free energy hypersurfaces. The reaction coordinates are graphed from atomic and molecular components to define points, distances, vectors, angles, planes and combinations thereof. The resultant free energy surfaces that are a function of the distance, angles, planes, and so on, can represent molecular mechanisms in reduced dimensions from the component atomic Cartesian coordinate degrees of freedom. The FEARCF library can be interfaced with any molecular package. Here, we demonstrate the link to NWChem to compute a hyperdimensional DFT (aug‐cc‐pVDZ basis set and X3LYP exchange correlation functionals) free energy space of a water dimer. Analysis of the water dimer free energy hypervolume reveals that while the chain and cyclic hydrogen bonding configurations are located in stable minimum energy wells, the bifurcated hydrogen bond configuration is a gateway to instability and dimer dissociation.

## Graphical abstract

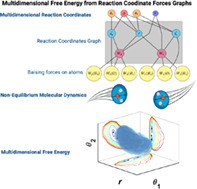

## 1 INTRODUCTION

The calculation of the free energy of a system involving molecular transitions relying on the redistribution of electrons is a computational challenge. The dynamics of hydrogen bonding in a water pair where the strong hydrogen bond donor and acceptor qualities of water include significant covalent character, is a case in point. Molecular dynamics methods relying on classical force fields have been used to describe condensed phase structure by computing spatial distribution functions^1^ and Laage et al. have shown that water reorientation occurs via the molecular jump mechanism.^2^, ^3^ To completely understand the phase equilibria of water it is necessary to compute the thermal properties, principally the free energy of association. A central requirement in the computation of free energy is rigorous sampling of the association, conformational, configurational or reaction space fundamental to the free energy landscape for the event(s) under investigation. There are several free energy methods that rely on classical force fields including Umbrella sampling,^4^ adaptive biasing potential^5^ and meta‐dynamics,^6^ all of which use a pre‐chosen analytical biasing potential to drive molecular systems from regions of low potential energy to regions of high potential energy. The process of choosing weighting function(s) presupposes knowledge of a mechanism for which the free energy landscape is then computed. By way of example, selecting the intermolecular distance between reacting molecules and then implementing a series of umbrella potentials along that coordinate, prevents the system from evolving freely and revealing the interplay of multiple possible reaction mechanisms.

The Free Energy from Adaptive Reaction Coordinate Forces (FEARCF)^7^, ^8^, ^9^ method instead uses numerically derived biasing forces to facilitate complete sampling of the potential energy landscape. A complex and rugged biasing potential is constructed from the density distribution of a molecular system resulting from the sites visited in MD runs. The density distribution is updated after each FEARCF iteration and reaction coordinate forces are computed that drives the system away from equilibrium low energy regions toward less probable high energy areas. The converged iteration results in an unbiased sampled landscape which categorizes it as a Flat Histogram^10^ method. The name implies a convergence toward a condition where the molecular system of interest is able to visit every possible state with equal probability.

Incorporating directional hydrogen bonding between water molecules in any molecular model is central to accurately simulating the natural phenomena. However, a dynamic account of electron redistribution in hydrogen bonding is only possible using quantum dynamics methods.^11^, ^12^ What remains outstanding is the computation of the free energy of association of water that accounts for electron redistribution during non‐Boltzmann dynamics. Previous reporting of the FEARCF method^7^, ^13^, ^14^ involved an implementation within the CHARMM molecular dynamics software which limited its scope to classical and semi‐empirical systems. FEARCF has now been packaged within an object‐oriented software library enabling its utilization within various molecular dynamics software packages including ones suited for quantum mechanical systems. Recently we reported a computationally efficient method that combines ab initio Hartree Fock dynamics (QSL: Quantum Supercharger Library)^15^, ^16^ through MD packages with a comprehensive multidimensional Free energy method (FEARCF)^7^, ^8^, ^9^ that could be applied to problems of this nature.^17^

A selection of a single reaction coordinate, *ξ*_1_ to compute the free energy for complex problems such as the hydrogen bonding water dimer is likely to result in the conflation of several events in the 1D energy well. Constructing several reaction coordinates (RCs) that are diverse in geometry (*ξ*_1_ = *r*, *ξ*_2_ = *θ*, *ξ*_3_ = *φ* etc.) representing distance between points, angles between vectors, angles between planes, and so on, to map out multiple dimensions in free energy space is a computational challenge. The numerical approach of FEARCF avoids the complex Jacobian corrections required by other free energy methods. In addition, the object‐oriented design of the FEARCF library enables a more intuitive and efficient means to calculate the atomic biasing forces. This can be best understood by the representation of sets of RCs as graphs.

The FEARCF approach is to express the complex relationship between the RCs and the atomic coordinates and forces on them in terms of graphs. Graphs are composed of elements referred to as vertices (nodes) where the relationship between the nodes are described by edges that connect the nodes. Complex combinatorial selections of multiples of these two objects can model a large variety of problems. Chemical graph theory was first applied to the discovery and classification of molecular isomers, particularly hydrocarbons, using trees to represent molecular topologies.^18^, ^19^, ^20^ More recently other applications have been developed including using graphs to assign unknown spectral resonances in NMR data,^21^ discovery of new reaction mechanims^22^ and even predicting new reactions entirely.^23^

Here we report the use of graphs that reduce the complexity of defining nontrivial RCs and their role in easily combining multiple RCs to efficiently compute high dimension free energy landscapes. The library implementation of the FEARCF method that facilitates an interface with classical and quantum molecular dynamics packages is illustrated in an interface with CHARMM and NWChem to produce classical and quantum free energy hypervolumes (FEVs). By way of illustration the mechanism of association and dissociation of hydrogen bonds in a water dimer is discovered from the multidimensional DFT FEVs using NWChem/FEARCF computations.

## 2 METHODS

### 2.1 FEARCF method

Previously we have detailed the FEARCF as a flat histogram method^8^, ^9^, ^24^ that produces a converged free energy surface enabling equal sampling in molecular and atomic configurational and conformational space. Equal sampling for a set of RCs (*ξ*) requires that the summed effect of the system potential *W*(*ξ*) and the biasing potential *U*(*ξ*) must be a constant. For the sake of simplicity we can choose this constant to be 0. To reach convergence the condition:

(1) $$W\left(\xi \right)+U\left(\xi \right)=0$$

must be satisfied giving the ideal biasing potential of *U*(*ξ*) = −*W*(*ξ*), even though initially *W*(*ξ*) is not known. To discover the form of *W*(*ξ*) it is convenient to start with *U*_1_(*ξ*) = 0. Using a zero bias, a simulation of the molecular system produces a probability density *P*_1_(*ξ*). The Boltzmann relationship

(2) $$W_{1}\left(\xi \right)=-k_{B}T\ln P_{1}\left(\xi \right)\;$$

produces the first estimation of the system potential *W*_1_(*ξ*), from which the next estimate of the ideal biasing potential is computed

(3) $$U_{2}\left(\xi \right)=-W_{1}\left(\xi \right)=k_{B}T\ln P_{1}\left(\xi \right).\;$$

This updated estimate of the biasing potential is then used to run a second iteration of the system from which another probability density is extracted, however the new probability density is that of the biased simulation of the system, that is, $P_{2}^{\prime }$(*ξ*). The unbiased probability density *P*_2_(*ξ*) can be recovered since the biasing potential *U*_2_(*ξ*) is known because it was used to produce the biased probability density

(4) $$P_{2}\left(\xi \right)=CP_{2}^{\prime }\left(\xi \right)\exp\;\frac{U_{2}\left(\xi \right)}{k_{B}T},$$

where *C* is a normalization constant. With this new updated unbiased probability density, a new biasing potential *U*_3_(*ξ*) can be created using the relation shown in Equation (3). This process is continued iteratively until a roughly equal sampling occurs with a small ratio between high and low energy regions making it possible for the system to diffuse about the landscape freely. When this is the case, a close approximation of the systems potential *W*(*ξ*) has been discovered. To speed up this process, the weighted histogram analysis method (WHAM)^25^ is used to combine the result of multiple simulations run at each iteration. This embarrassingly parallel approach allows for a much faster approach to convergence than a serial simulation computation can achieve.

The manner in which the biasing potential *U*(*ξ*) is used to calculate individual biasing forces on each of the atoms used to define *ξ* is through a simple application of the properties of conserved potentials, especially the relation that states that the force is the gradient of the scalar potential, that is, $\vec{F}=-\nabla U$. The gradient is presented in terms of Cartesian coordinates however in FEARCF the potential is defined in terms of *ξ*. To resolve this discrepancy, we consider the chain rule.

(5) $$\nabla U\left(\xi \right)=\frac{\mathrm{dU}\left(\xi \right)}{d\vec{x}}=\frac{\partial U\left(\xi \right)}{\partial \xi }\frac{\partial \xi }{\partial \vec{x}}.$$

The first term on the right hand side is the gradient of the biasing potential which can be numerically approximated using interpolation schemes such as cubic splining. The second term is the partial derivative of the RCs in terms of the Cartesian coordinates of the atoms used to define *ξ*. In the case of multiple dimensions this term is split into several partial derivatives which have known analytical solutions and are computable.^26^, ^27^ This separation of the atomic forces into a product of partial derivatives is the rationale for representing RCs as graphs. The FEARCF library's use of objects aids in the efficiency and flexibility of computing free energies as a combination of points, distances, planes, vectors, or angles.

### 2.2 FEARCF library

The FEARCF library written in Fortran 90 is composed primarily of several defined modules. These modules range from containing common math functions and expressions that are called repeatedly by other modules, to the complex equations needed to perform both cubic and B‐spline interpolation. The central module executes variable definition and calculation functions that are needed to construct the RC, as well as transforms potentials in terms of RCs to atomic potentials. These objects are essential components that map to the nodes and edges of the RC graphs.

**FIGURE 1:**
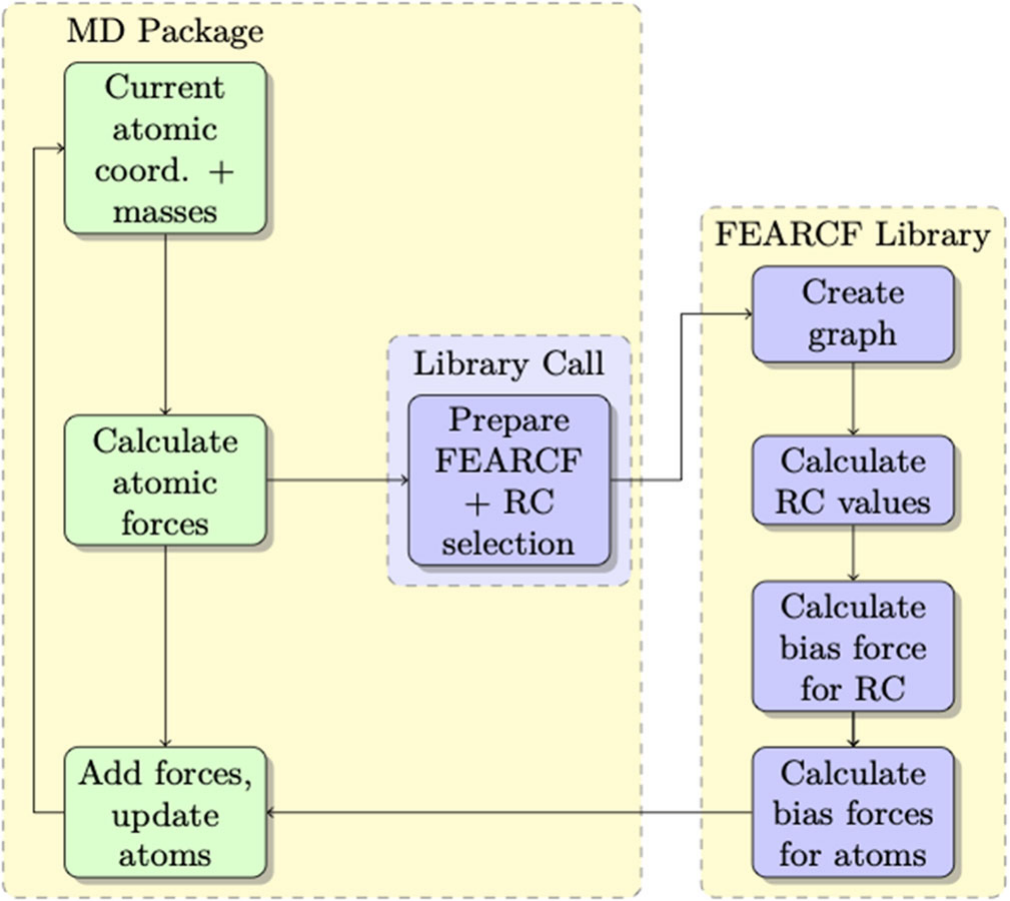
Illustration of linking the FEARCF library to a molecular dynamics package

A second significant module translates atomic coordinates, masses and forces data provided by the MD package into FEARCF syntax. The force and position data is amended as described above and directed back to the MD package. A library call to FEARCF is placed inside the MD package, typically at the end of the step update section as is illustrated in Figure 1.

The QMD implementation in NWChem has been previously reported.^28^ Briefly it has the functionality to perform ab initio molecular dynamics from methods including Hartree–Fock (HF), Density‐Functional Theory (DFT) and Møller–Plesset perturbation (MP2). This is achieved with a Born‐Oppenheimer approximation treatment of the nuclei, and modeling the electronic structure using Gaussian basis functions. In the case of DFT where the Kohn–Sham equations are given as

(6) $$\left(-\frac{1}{2}\nabla^{2}+v^{C}\left(\vec{r}\right)\right)\psi_{i}\left(\vec{r}\right)+\int v^{\mathrm{XC}}\left(\vec{r},{\vec{r}}^{\prime }\right)\psi_{i}\left({\vec{r}}^{\prime }\right)d{\vec{r}}^{\prime }=\epsilon_{i}\psi_{i}\left(\vec{r}\right),$$

where the nuclei are represented in the $v^{C}\left(\vec{r}\right)$ term as well as the electronic contributions to the Coulomb potential

(7) $$v^{C}\left(\vec{r}\right)=-\sum_{i}\frac{Z_{i}}{\left|{\vec{R}}_{i}-\vec{r}\right|}+\int \frac{\rho \left(\vec{r},\vec{r}\right)}{\left|\vec{r}-{\vec{r}}^{\prime }\right|}\;d{\vec{r}}^{\prime }.$$

The nuclei positions ${\vec{R}}_{i}$ are updated using the Velocity‐Verlet algorithm while the electronic structure $\rho \left(\vec{r},\vec{r}\right)$ is updated by solving the Schrödinger equation consistent with DFT theory.^29^ Through the FEARCF interface a biasing force is applied to each atom included in the graph definition and integrated into the NWChem force routine as is illustrated in Figure 1 prior to the start of the Velocity‐Verlet updating the nuclei positions. This separation of the nuclei and electronic motion is ideal since the graphs are constructed using the nuclei positions as the first set of nodes, meaning that the FEARCF biasing force can be seamlessly applied to the atomic nuclei without interfering in the electronic structure computation. This enables an interrogation of the role of molecular electron density in non‐Boltzmann state configurations. To achieve a canonical ensemble thermostats such as Langevin, Berendsen, and simple velocity scaling are used.

### 2.3 Graph Theory applied to reaction coordinates and biasing forces

The graphs constructed in FEARCF show in detail the relationships between the *ξ*_*i*_ and the atomic coordinates from which they are ultimately derived. The nodes represent several types of elements (atoms, vectors, planes, angles, etc.) while the edges represent the subroutines that both calculate the quantities themselves, as well calculate the partial derivative terms needed for the biasing force. In this way the constructed RCs can be represented as directed, colored graphs although an explicit inclusion of directed edges is not incorporated since every edge is implicitly bidirectional.

**FIGURE 2:**
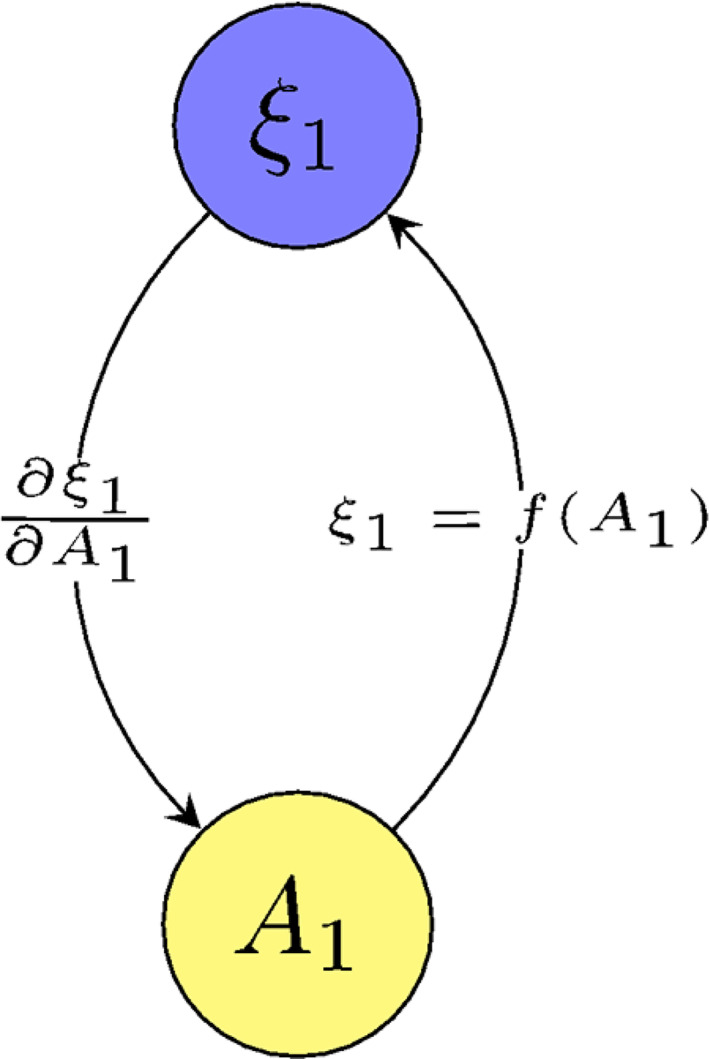
Simple graph illustrating basic function of a graph in the FEARCF library, with information being exchanged between an atom *A*_1_ and a reaction coordinate *ξ*_1_

To demonstrate how graphs can show this kind of relationship, a simple graph (Figure 2) is constructed between a single atom (*A*_1_) and a RC (*ξ*_1_) that has an explicit dependency on *A*_1_. Following this *ξ*_1_ is used to define a free energy *W*(*ξ*_1_). To illustrate that the nodes of the graph represent a variety of elements, the nodes are depicted as different color nodes. They share an edge which represents the potential flow of information (via subroutines) between them. For clarity, this edge is be split in two to represent the two types of information transfers namely calculation of the value of *ξ*_1_ as a function of *A*_1_, and the propagation of the biasing force via a product of partial derivatives, that is, $\frac{\partial W\left(\xi_{1}\right)}{\partial \xi_{1}}\frac{\partial \xi_{1}}{\partial A_{1}}$.

At every integration step of a FEARCF simulation the values of the chosen RCs need to be calculated from the atomic positions. To do this each chosen RC node retrieves information from the nodes, a level below, that are used to define it. If those nodes do not represent atoms, they in turn retrieve information from the nodes a further level below, that were used to define them. This creates a chain of information retrieval that terminates in the nodes representing the atoms of the system. The atomic coordinates are provided by the FEARCF interfaced MD package. From the atomic coordinates, all other nodes are defined and computed culminating in the RC values.

Once all RC values are known, the cubic or B‐spline interpolation subroutine is used to calculate the gradient, in particular the partial derivatives, of the free energy volume at these *ξ*_*i*_ values. The gradient partial derivatives received from the interpolation subroutine for each chosen *ξ* is sent to the nodes used to define it. These nodes then calculate the partial derivative term required to transform the received derivative to be in terms of the variable each node represents. These nodes then in turn send this derivative to the nodes used to define them. This is repeated in a chain‐like manner until the nodes that represent the atoms have accumulated all the partial derivatives needed for the biasing force on each atom (see Equation (5)). The atomic biasing forces are then added to the forces calculated by the MD package interfaced with the FEARCF library in order to update the atomic velocities and positions using both the system and biasing potential.

An early graphing approach defining RCs was implemented in the CHARMM module RXNCOR and a limited precursor to FEARCF^30^ expanded on this to undertake QM/MM reactions relying on a pair of distance RCs in two dimensions used for umbrella sampling.^30^, ^31^, ^32^ The original implementation in CHARMM was limited by umbrella sampling that is not ideally suited to free energy simulations of more than two dimensions and further is not efficiently parallelizable. The limit to two dimensions is due to the analytical nature of the biasing potential used in umbrella sampling that requires increasingly complex corrections with increasing dimensionality. The early implementation in CHARMM of the adaptive umbrella sampling method which uses WHAM to combine results from multiple parallel simulations has not been developed further to date.

The FEARCF library rectifies these limitations by sourcing its biasing potential from a numerical potential, which does not rely on change of frame corrections. The library being located outside of MD packages such as CHARMM better enables efficient integration of WHAM for parallelization. The graphing approach uses an adjacency matrix A defined as *A* = [*a*_*ij*_], $a_{\mathrm{ij}}\in \left(0,1\right)$ if node *i* and *j* share an edge. A simple graph representing the RCs and its adjacency matrix is given as(8)

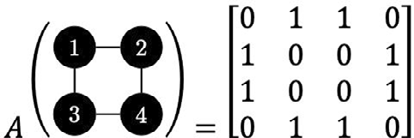




**FIGURE 3:**
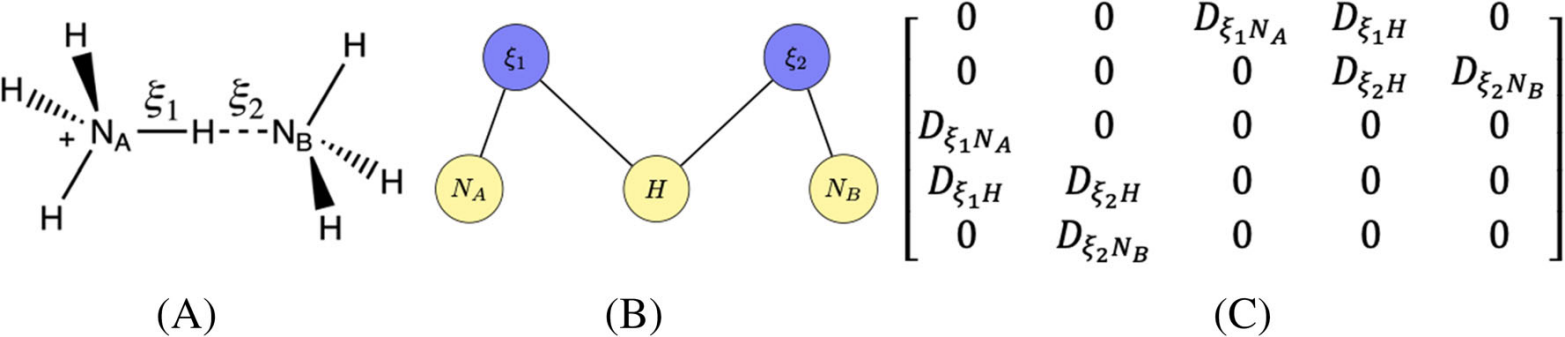
Representations of ammonium proton exchange reaction (A) molecular representation, (B) atoms defining *ξ*_1_ and *ξ*_2_ in colored graph, and (C) force computation from partial derivatives for each edge presented in derivative matrix

An associated matrix representing the forces, named here the derivative matrix *D*, which rather than simply having 1s representing the edges, they can be replaced by terms such $D_{\mathrm{ij}}=\frac{\partial i}{\partial j}$ which is the partial derivative of node *i* with respect to *j*. The atomic biasing forces can then be found by finding a path in the derivative matrix from each reaction coordinate to each connected atom node. The net atomic biasing force on an atom can then be expressed as the sum of the products of the derivatives along each path.^33^ An update of the previously computed proton exchange reaction [H_3_N—H—NH_3_]^30^ in CHARMM now defined using a graph representation of the reaction coordinates in FEARCF is shown in Figure 3.

The distance reaction coordinates (*ξ*_1_ and *ξ*_2_) as defined in terms of the molecular representation (Figure 3A) are simply represented in a two layer graph (Figure 3B). The force computation in FEARCF is shown in the derivative matrix (Figure 3C). The atomic biasing force dependencies and net force on the hydrogen being exchanged can be mathematically defined and computed (Equation (9)). Here *i* is an index over the set of reaction coordinates and *j* is an index over the edges that make up the path connecting the reaction coordinates to the atom.

(9) $$F_{H}=\sum_{i}\frac{\partial W}{\partial \xi_{i}}\prod_{j}D_{i,j}=\frac{\partial W}{\partial \xi_{1}}D_{\xi_{1}H}+\frac{\partial W}{\partial \xi_{2}}D_{\xi_{2}H}=\frac{\partial W}{\partial \xi_{1}}\frac{\partial \xi_{1}}{\partial H}+\frac{\partial W}{\partial \xi_{2}}\frac{\partial \xi_{2}}{\partial H}.\;$$

The subroutines used to calculate partial derivatives are defined and represented as edges in the FEARCF library. Theoretically, these routines are needed for every node type pairing; however, there are mathematical conditions that guide the combinations of nodes that define new reaction coordinate nodes (e.g., an angle node is only defined by two vector nodes). This significantly reduces the number of subroutines needed. With this construct of nodes and edges any multidimensional graph representing a combination of several geometric and/or conformational and/or configurational molecular properties is calculable by the FEARCF library. This is only possible because of the library's object‐oriented structure and the representation of every *ξ*_*i*_ as graphs.

### 2.4 Simulation conditions

All classical MD simulations are run in CHARMM 42b2^31^ using the CHARMM force field, run at 300 K in periodic boxes of size 12 Å each side, with non‐bonded interactions cut‐off at 14 Å, with the Velocity‐Verlet integrator in a Nose–Hoover ensemble thermostat with 1 heat bath, a tau value of 0.1 ps and 10 sub‐steps per time step. Each simulation is run with a 1 fs time step, with no equilibrium steps and a unique set of four 7 digit random seed numbers.

All quantum MD simulations are run in NWChem 7.0.2^29^ compiled with OpenMPI using the Gaussian basis ab initio QMD module, using the DFT level of theory with a Langevin thermostat with a friction value of 0.001. All simulations took place within a cubic cavity of 12 Å which applies a spring force to any atom straying outside the cavity with a spring constant of 5.5083 × 10^−6^ $E_{h}/a_{o}^{2}$. All simulations are run at 298.15 K, with a 1 fs time step, no equilibrium steps and a unique 7 digit random seed number. NWChem enables the parallelization of each simulation using a combination of the Global Array toolkit and MPI to enable efficient data transfer and load balancing during multi‐core simulations.

## 3 RESULTS AND DISCUSSION

Here we demonstrate the effective graphing structure of the FEARCF library applied to the interplay of hydrogen bonding in a water dimer. The value of multidimensional FEVs is illustrated by first computing a simple 1D free energy curve, followed by a 2D free energy surface. These computations have previously been performed using classical^34^, ^35^ and quantum^36^ methods. In these low dimension free energy computations each point on the line and surface is made up of an ensemble of configurations that are a conflation of linear chain (C), cyclic (Cy) or bifurcate hydrogen bonds making it impossible to distinguish between them and their contributions to the free energy of the system.

### 3.1 Free energy graph based computation

Free energy is an essential component needed to understand molecular mechanisms. The value of multidimensional reaction dynamics using FEARCF's have been shown for complex enzymatic catalyzed reactions.^17^, ^37^ Further the hypersurface FEVs using FEARCF for the TIP water system illustrated the sensitive nature of FEVs showing that they can be used to distinguish between TIP3P, TIP4P, and TIP5P models.^14^ However we did point out that a true mechanism of intermolecular hydrogen bond exchange was not possible using only classical methods. Here, the free energy volumes are computed for a classical water dimer model only to illustrate (i) the graphing approach to reaction coordinate force computation and (ii) demonstrate the flat histogram convergence of FEARCF. The mechanism of hydrogen bond association and dissociation are derived exclusively from an analysis of the QMD FEVs.

#### 3.1.1 Reaction coordinate and convergence of the 1D FE water dimer

While water is a simple molecule the collective structures and properties in bulk liquid and solid are complex. In hexagonal ice, a water molecule forms a tetrahedral structure with its four closest neighboring water molecules where it participates as a donor in two hydrogen bonds and as an acceptor in two more hydrogen bonds. This local structure is retained in the water solvent form.^38^ A key reason for this is the intermolecular hydrogen bonds that form between water molecules. A single water molecule is able to form up to four hydrogen bonds at once, a large number for a molecule of such low molecular weight. These numerous hydrogen bonds are the reason for water's high boiling point and greater density as a liquid compared to a solid.

**FIGURE 4:**
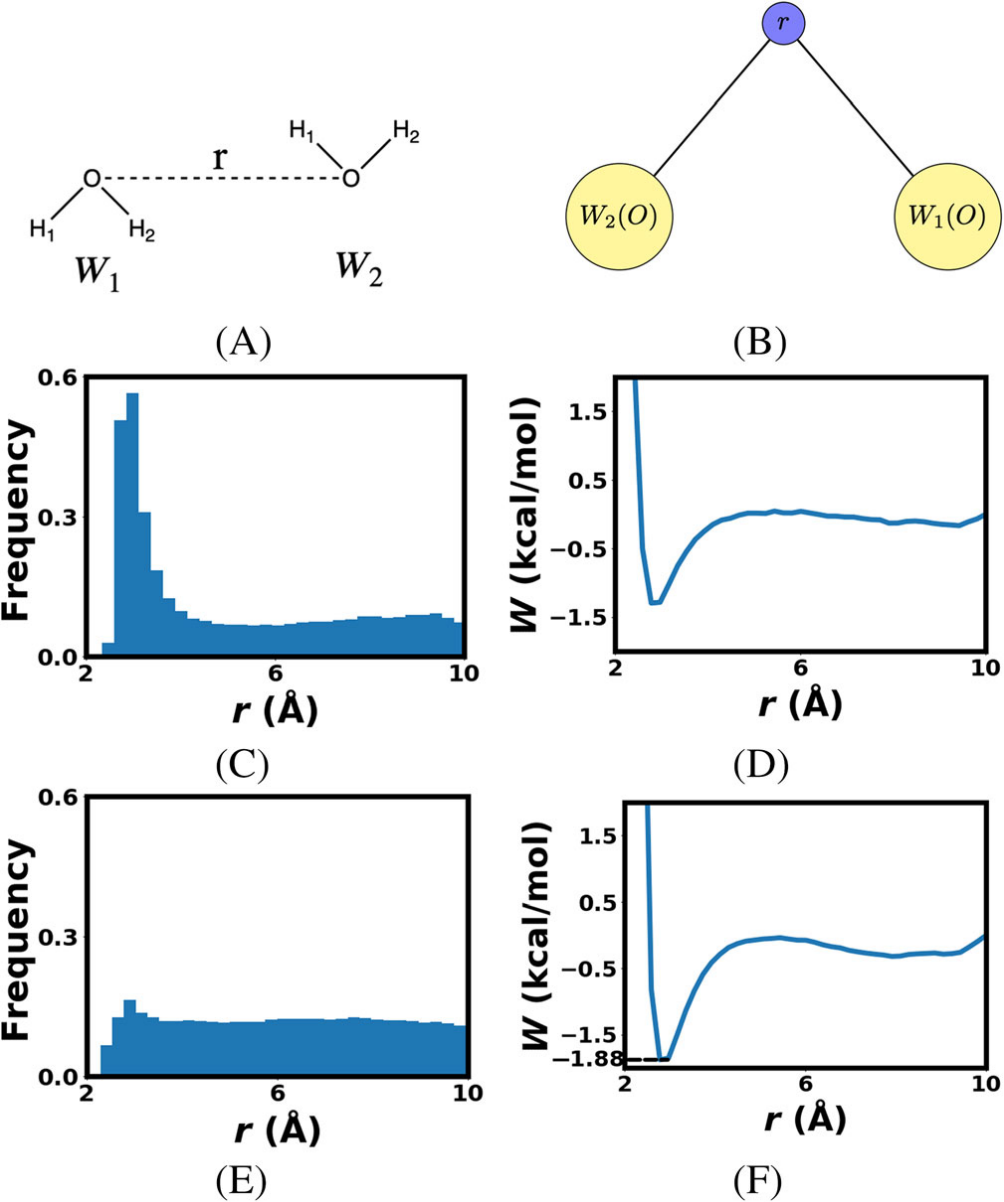
(A) 1D reaction coordinate for 1D water dimer system, (B) 1D water dimer RC graph representation with yellow nodes for atoms, blue/purple node for distance, (C) histogram showing sampling of 1st iteration, (D) FE surface of 1st iteration, (E) histogram showing sampling of 15th iteration, and (F) FE surface of 15th iteration with annotated minimum free energy value

First let us consider one reaction coordinate, namely the well‐studied scalar distance between the oxygen atoms for the water dimer system (Figure 4A) along with the graph that represents this reaction coordinate (Figure 4B). The oxygen atoms are represented as points through which a distance between the two waters are defined. This distance is the reaction coordinate. The flow of information via the edges in Figure 4B where the value of node r is simply given by $r=\sqrt{{\left({\vec{x}}_{W_{2}\left(O\right)}-{\vec{x}}_{W_{1}\left(O\right)}\right)}^{2}}$. The atomic biasing forces computed through the addition of the derivative in terms of r, is the unit vector $\hat{r}$. This is given in Equation (10).

(10) $$\begin{array}{c} {\vec{F}}_{r}=-\frac{\partial V}{\partial r}\hat{r.}\\ {\vec{F}}_{W_{1}}={\vec{F}}_{r}.\\ {\vec{F}}_{W_{2}}=-{\vec{F}}_{r}. \end{array}$$

The 1D classical Water Dimer FEARCF simulation is run for 50 ps each iteration, with 128 simultaneous simulations in each iteration, each simulation run with a single core.

The sampling along *ξ* = r during the first FEARCF iteration where the biasing force = 0 is centered at 3 Å with the ratio of highest to lowest sampling being 19.3:1 (Figure 4C). After the 15th FEARCF iteration (Figure 4E) the sampling along r converges toward a flat histogram with a high to low sampling ratio of 2.5:1. The minimum free energy occurs at r = 2.9 Å. Considering that the covalent bond length between the oxygen and the hydrogen in the TIP3P model is 0.9572 Å the minimum well is presumed to be a combination of hydrogen bond configurations with average length 1.9428 Å.

The magnitude of the free energy well at −1.88 kcal/mol does agree with the hydrogen bond strength being between that of Van Der Waals forces and Covalent/Ionic bonds. However, this one dimensional FEV gives us no insight into the actual orientational preference of water dimer hydrogen bonds.

#### 3.1.2 Reaction coordinates and convergence of the 2‐D FE water dimer surface

**FIGURE 5:**
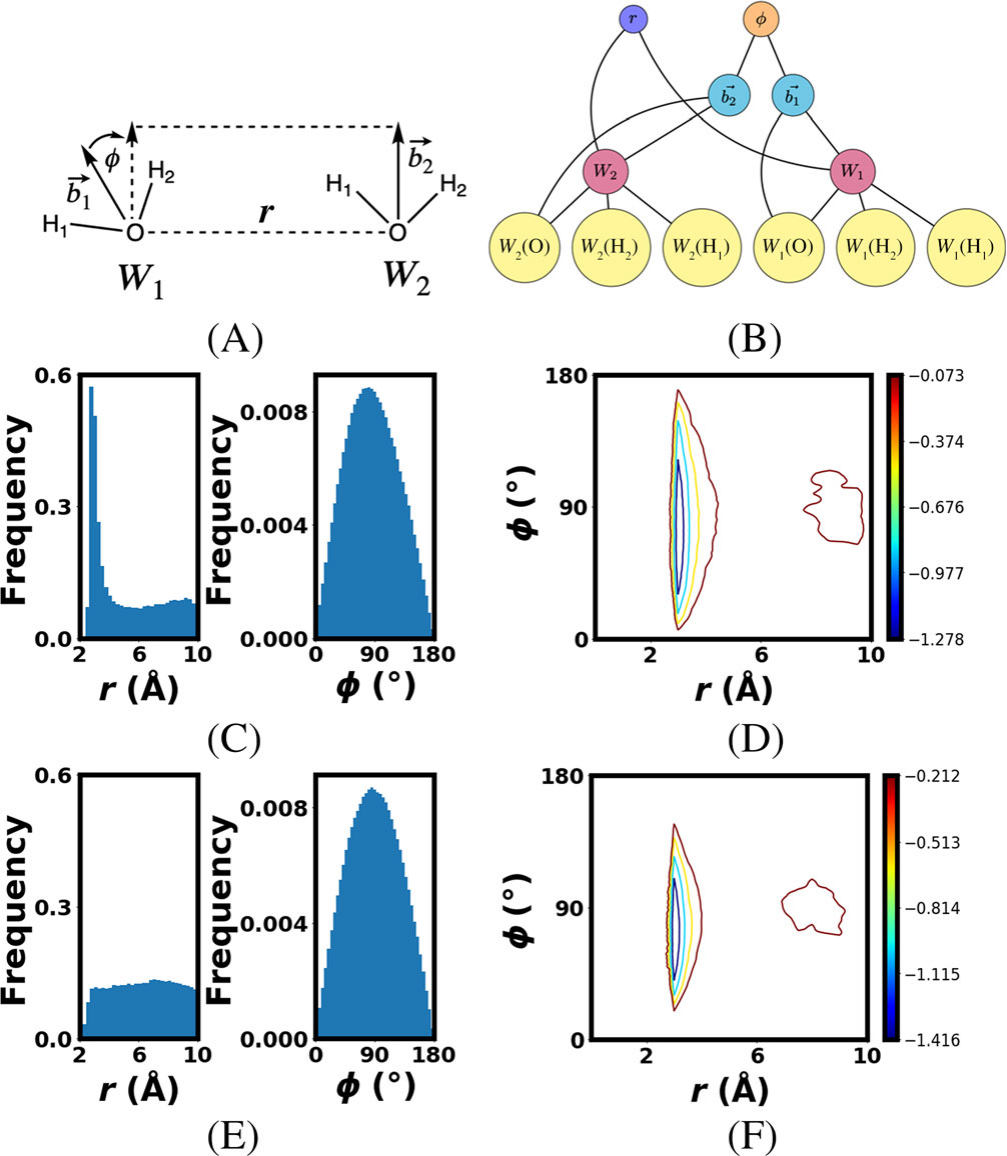
(A) 2D reaction coordinate for 2D water dimer system and (B) 2D water dimer RC graph representation with yellow nodes for atoms, blue node for distance, red nodes for center of masses, cyan nodes for vectors and orange node for angle, (C) Histograms showing sampling of 1st iteration, (D) FE surface of 1st iteration with contours at ½ *kT* intervals, (E) Histograms showing sampling of 12th iteration, and (F) FE surface of 12th iteration with contours at ½ *kT* intervals

Next we show another common setup for investigating water dimer interactions where two reaction coordinates (Figure 5A) include a distance (between the center of mass of each water molecule) and an angle (between the two dipole moments of each water molecule). An illustration of the graphing construct for these reaction coordinates is given in Figure 5B. Since the distance is now between centers of mass, all of the atoms in the system are represented as points. The oxygen atom and two hydrogens of a water is needed to define the center of mass *W*_*i*_. This reaction coordinate is the scalar distance *ξ*_1_ = *r* between the centers of mass. The second reaction coordinate requires a definition of the dipole moment vector $\vec{b_{i}}$ for each water molecule. This is done by simply defining a vector that points from the oxygen atom to the center of mass for each water molecule. While these are not actually the true dipole moment vectors, they are parallel to them which allows us to get the correct value for our angle reaction coordinate *φ*.

To translate the data via edges shown in Figure 5B $r=\sqrt{{\left({\vec{x}}_{W_{2}}-{\vec{x}}_{W_{1}}\right)}^{2}}$ and $\phi =\arccos\left({\vec{b}}_{1}\cdot {\vec{b}}_{2}/\left|{\vec{b}}_{1}\cdot {\vec{b}}_{2}\right|\right)\;$ are needed for the *ξ* nodes. In the case of the atomic biasing forces a similar approach for *r* as in Equation (10) is used. However, for ϕ the forces for the nodes are given in Equation (11).

$${\vec{F}}_{W_{1}}=-\frac{\partial V}{\partial \phi }\frac{\partial \phi }{\partial {\vec{x}}_{W_{1}}},\;{\vec{F}}_{W_{1}\left(O\right)}=-\frac{\partial V}{\partial \phi }\frac{\partial \phi }{\partial {\vec{x}}_{W_{1}\left(O\right)}},\;{\vec{F}}_{W_{2}}=-\frac{\partial V}{\partial \phi }\frac{\partial \phi }{\partial {\vec{x}}_{W_{2}}},\;{\vec{F}}_{W_{2}\left(O\right)}=-\frac{\partial V}{\partial \phi }\frac{\partial \phi }{\partial {\vec{x}}_{W_{2}\left(O\right)}}\;,$$

$$\frac{\partial \phi }{\partial {\vec{x}}_{W_{1}}}=-\frac{1}{\left|b_{1}\right|}\left({\hat{b}}_{1}\times \left({\hat{b}}_{2}\times {\hat{b}}_{1}\right)\right),\;\frac{\partial \phi }{\partial {\vec{x}}_{W_{2}}}=-\frac{1}{\left|b_{2}\right|}\left({\hat{b}}_{2}\times \left({\hat{b}}_{2}\times {\hat{b}}_{1}\right)\right),$$

(11) $$\;\frac{\partial \phi }{\partial {\vec{x}}_{W_{1}\left(O\right)}}=-\frac{\partial \phi }{\partial {\vec{x}}_{W_{1}}},\frac{\partial \phi }{\partial {\vec{x}}_{W_{2}\left(O\right)}}=-\frac{\partial \phi }{\partial {\vec{x}}_{W_{2}}}.\;$$

For the atom nodes used to define the center of masses, forces are simply weighted according to their atomic masses. The 2D classical water dimer FEARCF simulation is run for 50 ps each iteration, with 128 simultaneous simulations in each iteration, each simulation runs on a single core. The sampling for both *r* and ϕ (Figure 5C) for the first FEARCF iteration that has a zero biasing force has a high‐to‐low sampling ratio of (*ξ*_1_ = *r*; *ξ*_2_ = ϕ) is (8:1; 28:1) while after the convergence toward a flat histogram the ratio is (1.14:1; 26:1).

While the same minimum energy distance is observe (Figure 5F) as in the 1D case (Figure 4F) now the preference of the dipoles to orient themselves between 40° and 120°, that is, a preference for orthogonal configurations hints at hydrogen bonding configurations. Orthogonal dipole orientations more closely resemble linear chain compared with bifurcated hydrogen bonding. However, there is no single minima for the *φ* angle. This is consistent with an understanding that *φ* cannot distinguish between an orientation where the hydrogen bonding is donated from one water giving a bifurcated configuration or when the waters are laying alongside each other. As we have previously noted, dipole interactions are typically described by two degrees of freedom, that is, the separation of the centers and the angle between them. This is a sparse description and contains not much more information than *W*(*r*).^14^

#### 3.1.3 Reaction coordinates and convergence of the 4‐D FE water dimer hypersurface

**FIGURE 6:**
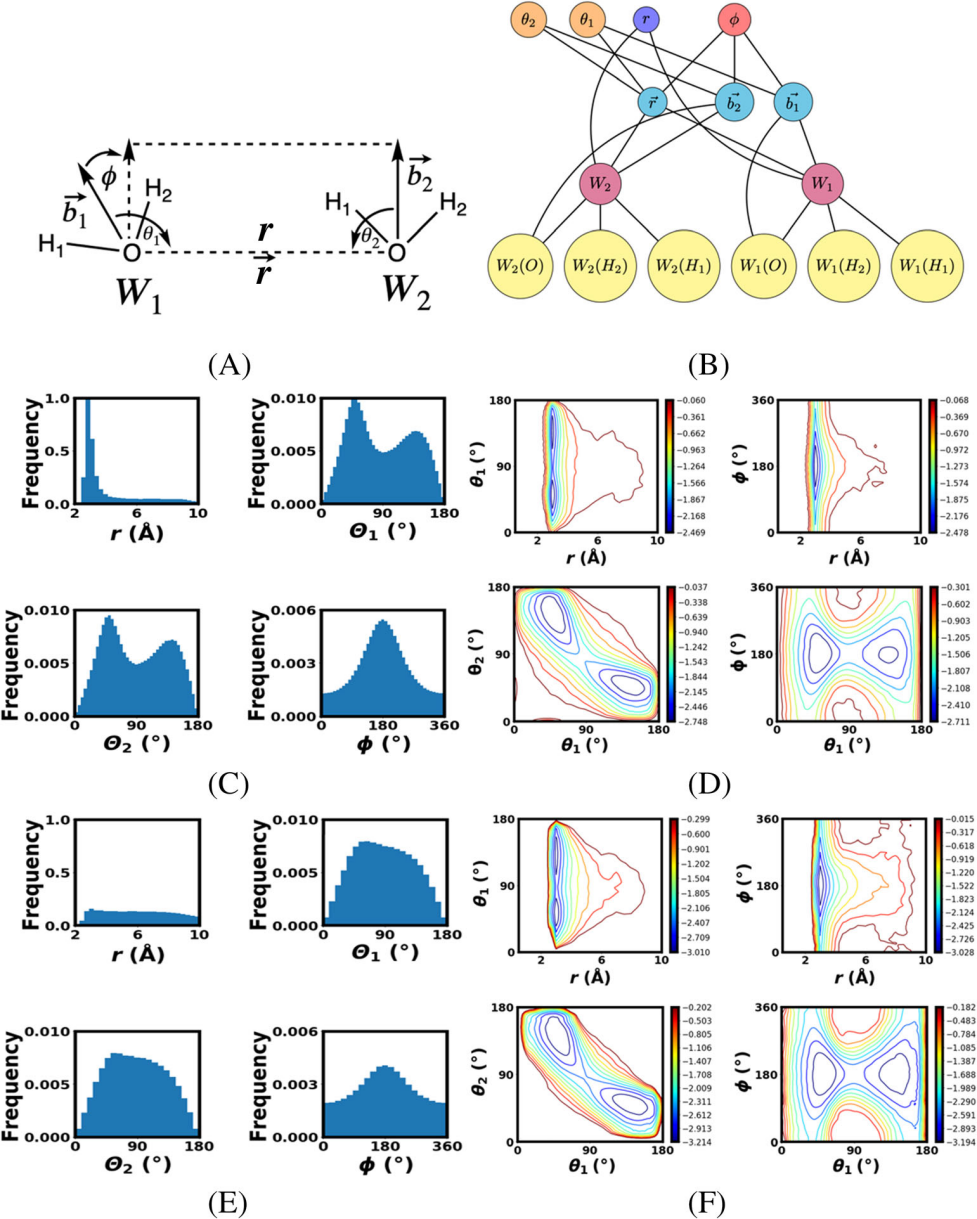
(A) 4D reaction coordinate definitions for 4D water dimer system, (B) 4D water dimer RC graph representation yellow nodes for atoms, blue/purple node for distance, red nodes for center of masses, cyan nodes for vectors, orange nodes for angles and red node for pucker angle, (C) histograms showing sampling of 1st iteration, (D) 2D Boltzmann averaged FE surfaces for 1st iteration with contours at intervals of ½ *kT* kcal/mol, (E) histograms showing sampling of 5th iteration, and (F) 2D Boltzmann averaged FE surfaces for 5th iteration with contours at intervals of ½ *kT* kcal/mol

Previously we concluded that four reaction coordinates was a useful description leading to the 4D *W*(*r, θ*_1_ *, θ*_2_, and *ϕ*).^14^ Here, the parameters are distance between centers of masses (*r*), the molecular vector angles (*θ*_1_ and *θ*_2_) and their relative orientation (*ϕ*) as shown in Figure 6A. It is similar to the 2D system with the same definition for *r* however, *φ* is now a dihedral angle. In addition we have defined angles between the dipole moment vectors $\vec{b_{i}}$ and the vector version $\vec{r}$ of our previously defined distance *r*. Constructing a graph that best represents these reaction coordinates is illustrated in Figure 6B. Again firstly all the atoms in the system are defined as points from which the center of masses *m* for each water molecule can be described. This alone defines the distance reaction coordinate *r* as well as its vector $\vec{r}$. Following this, the vectors parallel with the dipole moment vectors $\vec{b_{i}}$ can be described which in turn allows for a definition of the dihedral angle *φ* between. In addition two angles *θ*_1_ and *θ*_2_ between the dipole moment vectors $\vec{b_{i}}$ and the intermolecular vector $\vec{r}$ are needed.

The value for the *r* RC node in Figure 6B is the same as for the 2D case while $\theta_{1}=\arccos\frac{{\vec{b}}_{1}\cdot \vec{r}}{\left|{\vec{b}}_{1}\cdot \vec{r}\right|}\;$, $\theta_{2}=\arccos\;\frac{{\vec{b}}_{2}\cdot \vec{r}}{\left|{\vec{b}}_{2}\cdot \vec{r}\right|}\;$, and $\phi =\arccos\frac{\vec{m}\cdot \vec{n}}{\left|\vec{m}\cdot \vec{n}\right|}\;$ where $\vec{m}={\vec{b}}_{1}\times \vec{r}$ and $\vec{n}={\vec{b}}_{2}\times \vec{r}$ which are the normal to planes defined for each water molecule's dipole moment. The atomic biasing forces resulting from *r*, $\theta_{1}$, and $\theta_{2}$ are much the same for the 1D and 2D cases, however for the dihedral angle ϕ Equation (12) gives the forces sent via edges to the point nodes used to define the vector nodes $\vec{r}$, ${\vec{b}}_{1}$, and ${\vec{b}}_{2}$.

$${\vec{F}}_{W_{1}}=-\frac{\partial V}{\partial \phi }\left|r\right|\frac{\vec{m}}{{\left|m\right|}^{2}},{\vec{F}}_{W_{2}}=-\frac{\partial V}{\partial \phi }\left|r\right|\frac{\vec{n}}{{\left|n\right|}^{2}},$$

$${\vec{F}}_{W_{1}\left(O\right)}=-{\vec{F}}_{W_{1}}+\left(\frac{\vec{r}\cdot {\vec{b}}_{1}}{r^{2}}\right){\vec{F}}_{W_{1}}+\left(\frac{\vec{r}\cdot {\vec{b}}_{2}}{r^{2}}\right){\vec{F}}_{W_{2}},$$

(12) $${\vec{F}}_{W_{2}\left(O\right)}=-{\vec{F}}_{W_{2}}-\left(\frac{\vec{r}\cdot {\vec{b}}_{1}}{r^{2}}\right){\vec{F}}_{W_{1}}-\left(\frac{\vec{r}\cdot {\vec{b}}_{2}}{r^{2}}\right){\vec{F}}_{W_{2}}.$$

The 4D classical water dimer FEARCF simulation is run for 50 ps each iteration with 128 simultaneous simulations in each iteration; each simulation being run on a single core. The sampling for all *ξ*_*i*_ in the first FEARCF iteration with no biasing forces has (*ξ*_1_ = *r*; *ξ*_2_ = $\theta_{1}$; *ξ*_3_ = $\theta_{2}$; *ξ*_2_ = *φ*) a high‐to‐low ratio for the distance of (57.4:1) in Figure 6C while after the 5th FEARCF iteration approximate convergence was reached with high‐to‐low ratios of (1.8:1) in Figure 6E.

The preference for *φ* = 180° and *θ*_1_ = 52°/128° is due to 52° being half of the TIP3P parameter angle formed between the two hydrogen‐oxygen covalent bonds. At *θ*_1_ = 52° one of the hydrogen atoms of the first water is now in line with the vector $\vec{r}$ facing the oxygen of the second water, while one of the hydrogens of the second water is also in line with $\vec{r}\;$ but facing away from the other oxygen. Lastly *φ* = 180° indicates that the second hydrogen of each water molecule orient themselves away from each other due to electrostatic repulsion.

### 3.2 Water dimer DFT QMD free energy volumes

All ab initio water dimer simulations were run with 10 ps per iteration, 30 simultaneous simulations, with 8 cores for each simulation using the aug‐cc‐pVDZ basis set and the X3LYP exchange‐correlation functional. This is following Plumley and Dannenberg^39^ reporting that aug‐cc‐pVDZ basis set and the X3LYP exchange‐correlation functional reproduced similar binding energies to functionals with larger number of terms (like aug‐cc‐pVTZ) without CP‐corrections. In addition they found that the bond distances for O—H and O—O were better modeled unlike some small functionals that over or underestimate the bond length.

There is a clear difference between the classical 1D water dimer free energy compared to the 1D ab initio free energy. The TIP3P model has a minimum of −1.88 kcal/mol while ab initio has a minimum of −3.92 kcal/mol. This is due to the limitations of the classical CHARMM TIP3P forcefield which fails to capture the subtle nature of hydrogen bonds that relies on a reordering of electron density to represent strong hydrogen bonds that have significant covalent character.

**FIGURE 7:**
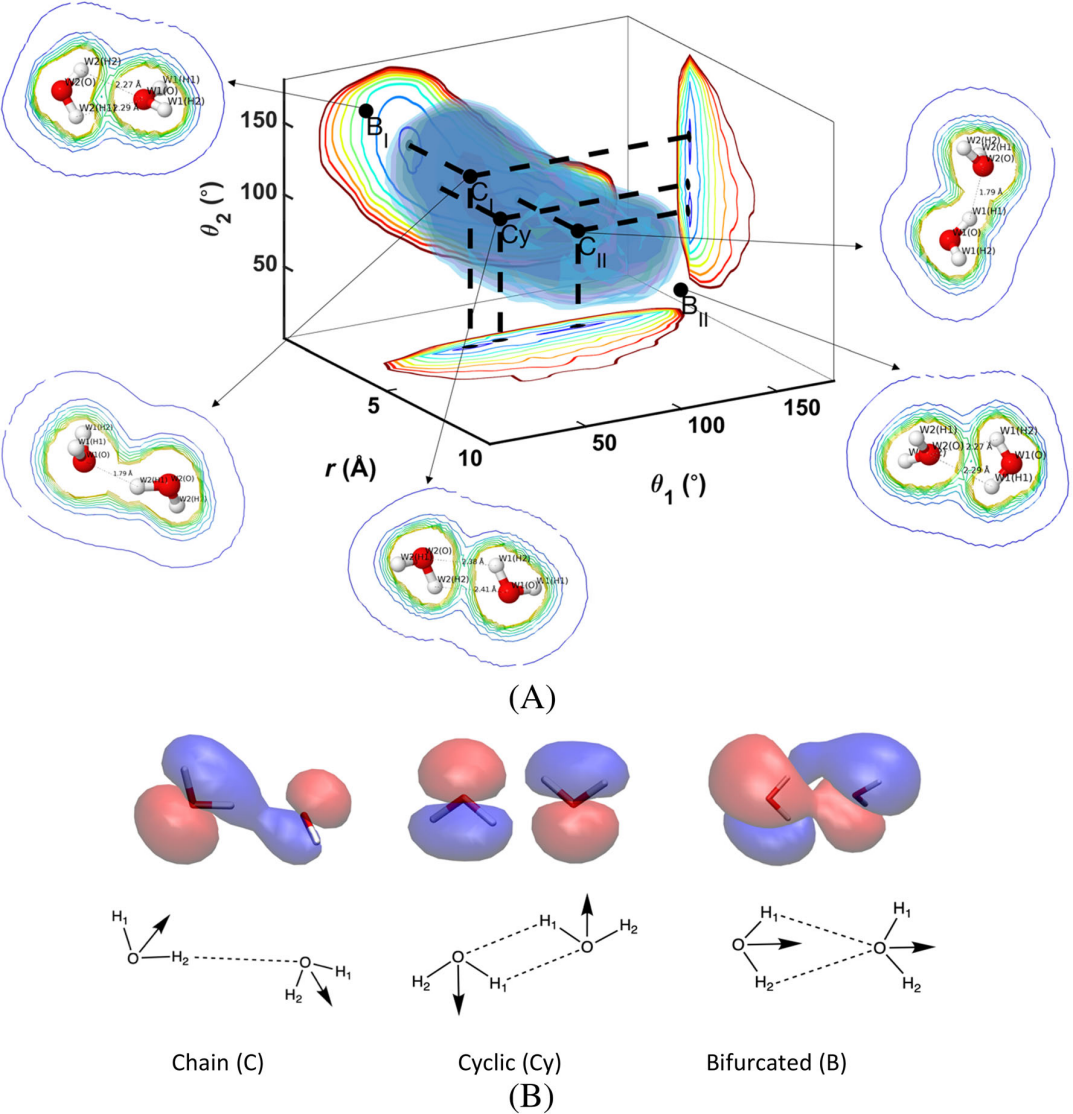
(A) 3D FEV for 30th FEARCF iteration with iso‐surfaces and contours, along with overlaid electron density contours at Chain, Cyclic, and Bifurcated configurations and (B) molecular orbital plot of 3*a*_1_ for proton donor and 1*b*_1_ for proton acceptor at 0.03 au for the 3 hydrogen bonding configurations with positive density in red and negative density in blue along with illustrations of each configuration

Previously Van Thiel et al.^40^ described three potential configurations for a water dimer to form hydrogen bonds that they named Chain (C), Cyclic (Cy), and Bifurcated (B) which are illustrated in Figure 7B. There is a minima well observed in the 4D ab initio water dimer FEV that comprises two configuration types C and Cy. Since the system is symmetrical two equivalent Chain configurations are seen C_I_ (*r* = 2.96 Å, *θ*_1_ = 65.53°, and *θ*_2_ = 118.33°) and C_II_ (*r* = 2.90 Å, *θ*_1_ = 122.61°, and *θ*_2_ = 66.23°) with Cy (*r* = 2.90 Å, *θ*_1_ = 81.87°, and *θ*_2_ = 84.50°) midway between the two. While the *r* minima value agrees with the classical value, the *θ*_1_ and *θ*_2_ minima values do not. This is due to the decreased rigidity of the ab initio model allowing for the water molecule to deform when strongly hydrogen bonded and there is electron density sharing between the hydrogen donor and the oxygen acceptor atoms. Overlayed in Figure 7A are electron density contour plots of C_I_, C_II_, C_y_, B_I_, and B_II_. The closest contours are at a density of 0.03 au where it is apparent that there is a sharing of electrons in the chain case (C_I_ and C_II_) that does not occur for C_y_ B_I_, or B_II_. This implies that the hydrogen bonds formed at C_I_ and C_II_ are not purely electrostatic in nature but also involve deformation of the electron clouds of the hydrogen bond donor and acceptor (Figure 7B and Table 1). While the C and Cy configurations are located in the *kT* minima well the B configurations are just outside of the 6 *kT* broad envelope of hydrogen bonded configurations. The geometric and electronic values for the hydrogen bonding configurations derived from the FEV and subsequent electronic analysis are summarized in Table 1.

**TABLE 1:** Differences in hydrogen bond length, electron density, and free energy for the three types of water dimer configurations

HB configuration	*r* (Å)	$\theta_{1}\;\left({}^{{}^{\circ}}\right)$	$\theta_{2}\;\left({}^{{}^{\circ}}\right)$	$\phi \;\left({}^{{}^{\circ}}\right)$	R_H(D)—O(A)_ (Å)	ρ (au)	∆G (kcal/mol)
C	2.96	65.5	118.3	183.1	1.94	0.022	−3.46
Cy	2.90	81.9	84.5	180.6	2.28	0.010	−3.24
B	2.70	172.4	7.9	–	2.27	0.006	10.04

**FIGURE 8:**
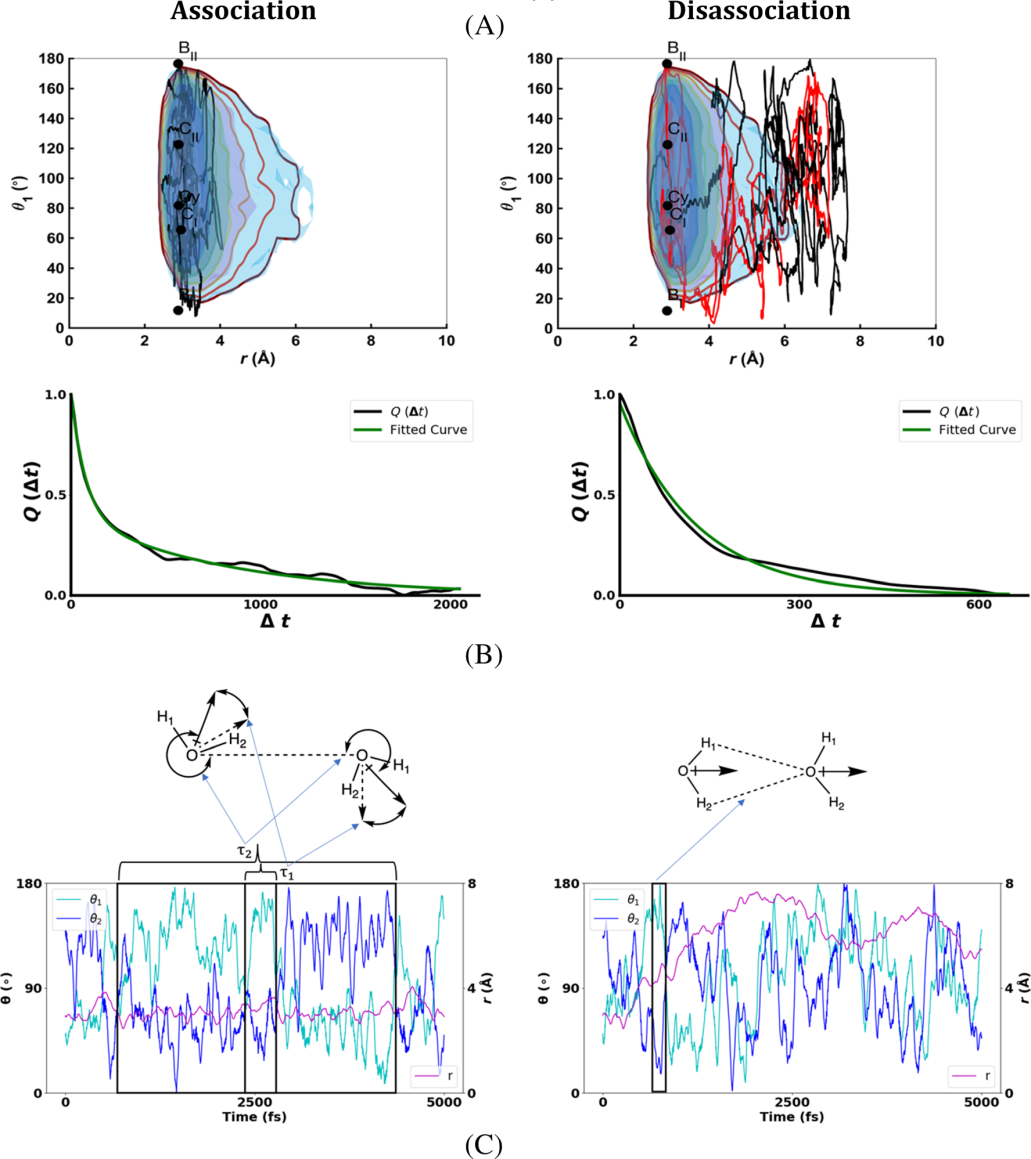
(A) Examples of 3 water dimer MD trajectories that either remain associated or become disassociated, (B) averaged dipole–dipole autocorrelation plots of 16 similar trajectories for each case, and (C) time series for the θ and *r* reaction coordinate values. The correlation and dissociation events are highlighted and the associated dimer configurations are illustrated

The 3D free energy hypersurfaces (Figure 7) provide clues to hydrogen bonding configurations of the water dimer as well as the shape of the ensemble of configurations. Now we attempt to uncover the mechanisms of the dimer association within the hydrogen bond minima well and the pathway from hydrogen bonding to dimer dissociation through a projection of trajectories onto the FEV (Figure 8A). In the case of association a trajectory that traverses within the 3 *kT* minima FE surface well appears to librate about a chain type configuration. It does this by passing through a cyclic configuration in‐between the two low energy chain configurations. The local minima labeled C_I_ (Figure 7) is defined by a hydrogen bond between *W*_1_(H_2_) and *W*_2_(O), with an overlap of the electron density highlighting the covalent nature of hydrogen bonds. Another local minima labeled C_II_ where *W*_2_(H2) is the hydrogen bond donor and *W*_1_(O) the acceptor, is the symmetric equivalent of C_I_. In between these two states is a pseudo transition state labeled as Cy where the two molecules are straddled alongside each other and no longer share electron clouds but where both molecules are hydrogen bond donors and acceptors. It is a mid‐point between C_I_ and C_II_.

The nature and extent of the water association is measured through the dipole–dipole correlation function given by $Q\left(t\right)=<\;D\left(0\right)∙D\left(t\right)\;>$ where *D*(*t*) is the total dipole moment of both water molecules at time t (left panel Figure 8B). To fit the $Q\left(t\right)$ decay 2 exponentials of the form $e^{-\left(t+x_{1}\right)/\tau_{1}}+e^{-\left(t+x_{2}\right)/\tau_{2}}$ were used. From this the relaxation times and fitting parameters ($\tau_{1}=75.7\pm 1.2,\;\tau_{2}=798.2\pm 6.4,\;x_{1}=36.93\pm 0.87,\;x_{2}=729\pm 11$) were found. The two exponential terms needed for the fit implies that within the association well there are two processes at play. The relaxation time is a measure of how fast a particular quantity is becoming uncorrelated. Trajectories from the association well were analyzed to unpack the two events (left panel Figure 8C). The faster relaxation time $\tau_{1}$ is due to the librational motion of both dipoles within the minima well about the angles of C_I_ (*θ*_1_ = 65.53° and *θ*_2_ = 118.33°) and C_II_ (*θ*_1_ = 122.61° and *θ*_2_ = 66.23°). This observation for an isolated water dimer is consistent with the well‐known phenomenon of water molecules libration in bulk liquid^41^, ^42^ indicating that water libration in hydrogen bonding configurations is an innate molecular property rather than a condensed phase induced phenomenon. The slower relaxation time $\tau_{2}$ is due to the periodic migration from one minima well C_I_/C_II_ area to another C_II_/C_I_ via the transition region Cy (Table 1).

Turning our attention to the process of hydrogen bond dissociation (right panel Figure 8A) we investigate trajectories to understand if there is a systematic molecular process of the hydrogen bond between the waters breaking while drifting apart. After passing the energy barrier of 6 *kT* = 3.55 kcal/mol, the water molecules remain more than 5 Å apart, are no longer hydrogen bonded and do not have an enthalpic means to re‐establish the hydrogen bond. The time correlation function can be fitted to single exponential function (right panel Figure 8B) of the form $e^{\frac{-\left(t+x\right)}{\tau }}$ where τ is the relaxation time. Upon fitting, the values are found to be τ=127.0±1, x=5.63±0.77. Analyzing the dissociation trajectories, two of which are projected onto the free energy surface (right panel in Figure 8A), shows that the exit out of the 6 *kT* hydrogen bond zone is made via a bifurcated hydrogen bonding configuration. A closer look at this through an examination of the time series of the θ and *r* RCs (right panel Figure 8C) confirms that the gateway out of the hydrogen bond zone of *r* > 4 Å is through the B_I_ and B_II_ configurations (red and black trajectories on right panel Figure 8A).

## 4 CONCLUSIONS

The implementation of graph theory to define complex reaction coordinates and draw detailed paths to atoms affected by the perturbations due to biasing forces has been detailed here. We showed that the convergence of a multidimensional free energy simulation as a flat histogram having a small sampling ratio between high energy to low energy events can be achieved. This free energy method has been reported previously as a set of algorithms linked to CHARMM. Here, we present a formalized library format for hyperdimensional free energy computations (FEARCF) that can be interfaced with several packages. The seamless link to NWChem presents the opportunity to produce advanced ab initio QMD multidimensional free energy hypersurfaces. We illustrate the value of this by investigating the hydrogen bonding nature of a water dimer by constructing the hyperdimensional DFT (aug‐cc‐pVDZ basis set and X3LYP exchange correlation functionals) free energy space of a water dimer. Through this we are able to understand that the association mechanisms of a water pair is grounded in two processes that govern the interplay between the chain and cyclic hydrogen bonding configurations. First, the correlated librational motion of both water dipoles that describes the dynamic C_I_/C_II_ configurations appears to be an innate molecular phenomenon that carries through to bulk water in the condensed phase. Second, there is a periodic interchange between the C_I_ and C_II_ configurations that is via the Cy configuration. In the case of hydrogen bond dissociation there appears to be an organized process that passes from the enthalpically stable dimer 6 *kT* configurational well into the random independent water dynamics via bifurcated hydrogen bonding. It is now possible to undertake ab initio hyperdimensional free energy investigations of molecular mechanisms through the interfacing of FEARCF with packages such as NWChem and so reduce the reliance on subjective parameterised QM models.

## Data Availability

The data that support the findings of this study are available from the corresponding author upon reasonable request.
